# The dynamic epitranscriptome: A to I editing modulates genetic information

**DOI:** 10.1007/s00412-015-0526-9

**Published:** 2015-07-07

**Authors:** Mansoureh Tajaddod, Michael F. Jantsch, Konstantin Licht

**Affiliations:** Department of Chromosome Biology, Max F. Perutz Laboratories, University of Vienna, Dr. Bohr Gasse 9/5, A-1030 Vienna, Austria; Department of Cell Biology, Center of Cell Biology and Anatomy, Medical University of Vienna, Schwarzspanierstrasse 17, A-1090 Vienna, Austria

## Abstract

Adenosine to inosine editing (A to I editing) is a cotranscriptional process that contributes to transcriptome complexity by deamination of adenosines to inosines. Initially, the impact of A to I editing has been described for coding targets in the nervous system. Here, A to I editing leads to recoding and changes of single amino acids since inosine is normally interpreted as guanosine by cellular machines. However, more recently, new roles for A to I editing have emerged: Editing was shown to influence splicing and is found massively in Alu elements. Moreover, A to I editing is required to modulate innate immunity. We summarize the multiple ways in which A to I editing generates transcriptome variability and highlight recent findings in the field.

## Introduction

The burst in genome sequencing has led to the surprising insight that genomic complexity does not reflect biological complexity. Instead, a similar number of genes can be found in organisms as different as nematodes, insects, or mammals (Szathmáry et al. 2001). However, proteomic complexity may well correlate with biological complexity and, therefore, may solve this paradox of modern biology (Licatalosi and Darnell 2010; Sabin Leah et al. 2013; Sie and Kuchka 2011). Proteomic complexity can be generated by protein modifications and transcript variations. Main mechanisms to introduce transcript variation are alternative splicing and RNA-editing. Indeed, alternative splicing is most abundant in mammalian neuronal tissues consistent with its role in generating transcript diversity (Barbosa-Morais et al. 2012). Similarly, the number of identified RNA-editing sites is highest in neuronal tissues both in mammals and invertebrates (Alon et al. 2015; Tariq and Jantsch 2012). While the impact of alternative splicing on transcriptome complexity is established since many years, the contribution of RNA-editing to transcriptome variation has only been studied systematically since the advent of deep-sequencing technologies.

In mammals, primarily two types of nucleotide deamination drive RNA-editing. Cytidine deamination by APOBECs leads to the conversion of cytidines to uridines (Blanc and Davidson 2003; Blanc and Davidson 2010). This type of RNA-editing was first believed to be rare but was recently shown to be abundant in noncoding parts of the transcriptome (Rosenberg et al. 2011). Adenosine to inosine deamination (A to I editing), on the other hand, is accomplished by adenosine deaminases acting on RNAs (ADARs). Two catalytically active ADARs, ADAR1 and ADAR2, are found in mammals (Kim et al. 1994; Maas et al. 1996; Melcher et al. 1996). Both enzymes bind double-stranded RNAs and have overlapping, yet distinct substrate specificities (Melcher et al. 1996). A third protein (ADAR3) apparently lacks enzymatic activity (Chen et al. 2000; Schneider et al. 2014).

**Fig. 1:**
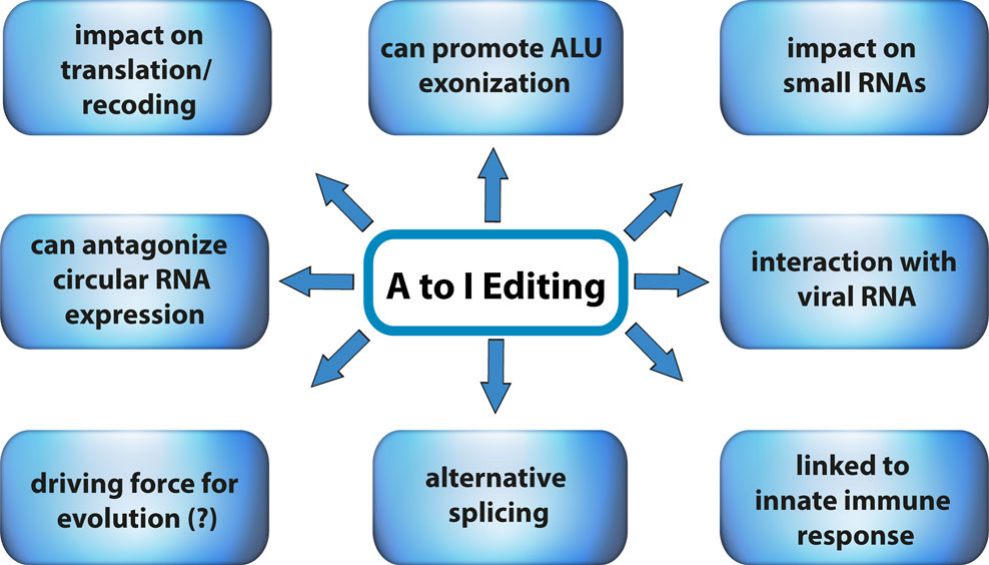
Adenosine to inosine RNA-editing affects the transcriptome in multiple ways. Effects of A to I editing range from recoding of amino acids, consequences for alternative splicing, and links to the innate immune response. For details, please refer to the text

In early bioinformatic approaches aimed at comparing transcriptomic and genomic data, about 100,000 A to I editing events have been discovered (Athanasiadis et al. 2004; Blow et al. 2004; Kim et al. 2004; Levanon et al. 2004; Levanon et al. 2005; Morse et al. 2002). With the advent of deep-sequencing technologies, the number of identified A to I editing events rapidly expanded to over two million edited sites in the human transcriptome (Li et al. 2009; Peng et al. 2012; Porath et al. 2014; Ramaswami et al. 2012; Ramaswami et al. 2013). The majority of these sites have been collected in two public databases: DARNED (http://darned.ucc.ie/) and RADAR (http://rnaedit.com/) (Kiran et al. 2013; Ramaswami and Li 2014). The most recent deep-sequencing study even suggests that over 100 million sites in the human transcriptome might be subjected to A to I editing, albeit many sites may only be targeted at very low levels (Bazak et al. 2014a). In mammals, a few hundred A to I editing events can recode mRNAs resulting in the translation of proteins that differ from their genomically encoded versions (Li et al. 2009). In contrast, the above-mentioned millions of editing events are largely located in the noncoding parts of mRNAs (Athanasiadis et al. 2004; Levanon et al. 2004). However, the biological consequences of editing events in noncoding parts of the transcriptome are only partly understood (Fig. 1). These range from RNA destabilization via inosine-specific cleavage, over changes in the folding of RNA, to inosine-dependent suppression of immune responses (Mannion et al. 2014; Vitali and Scadden 2010).

In this review, we will focus on different aspects of A to I editing and its impact on mammalian transcriptomes. Starting with a brief overview of the different editing enzymes, we continue with a comparison of specific and promiscuous editing as well as editing in coding substrates and repetitive elements. We will also focus on the conservation of editing, the coupling of editing and splicing, the regulation of A to I editing, and finally we briefly highlight quite recent findings on the involvement of A to I editing in the innate immune signaling and the antagonistic role of ADAR1 in circular RNA generation. For the impact of A to I editing on small RNAs such as miRNAs, we would like to direct the reader to the following reviews: Hundley and Bass (2010), Nigita et al. (2015), and Nishikura (2010).

## The ADAR class of enzymes

In mammals, two catalytically active ADAR enzymes are known: ADAR1 and ADAR2 (ADARB1). ADAR1 is ubiquitously expressed. Mice deficient of ADAR1 die between embryonic days 11.5 and 12.5, apparently due to hematopoietic defects and widespread apoptosis presumably induced by massive interferon signaling (Hartner et al. 2004; Hartner et al. 2009; Vitali and Scadden 2010; Wang et al. 2004). Mice lacking ADAR2 die from seizures within 3 weeks after birth (Higuchi et al. 2000). Interestingly, this dramatic phenotype can be rescued by the expression of a pre-edited allele encoding glutamate receptor subunit 2 (*Gria2*), suggesting that *Gria2* RNA may be the major substrate of ADAR2.

For ADAR3, a third ADAR protein, no catalytic activity has been detected to date, and expression was only seen in the brain (Chen et al. 2000; Schneider et al. 2014). In contrast, ADAR1 and ADAR2 are expressed in a wide range of tissues. Two isoforms of ADAR1 are known: ADAR1-p150 (150 kDa in size) has an interferon inducible promoter, whereas ADAR1-p110 (110 kDa) is constitutively expressed (George and Samuel 1999; Patterson and Samuel 1995; Patterson et al. 1995).

**Fig. 2:**
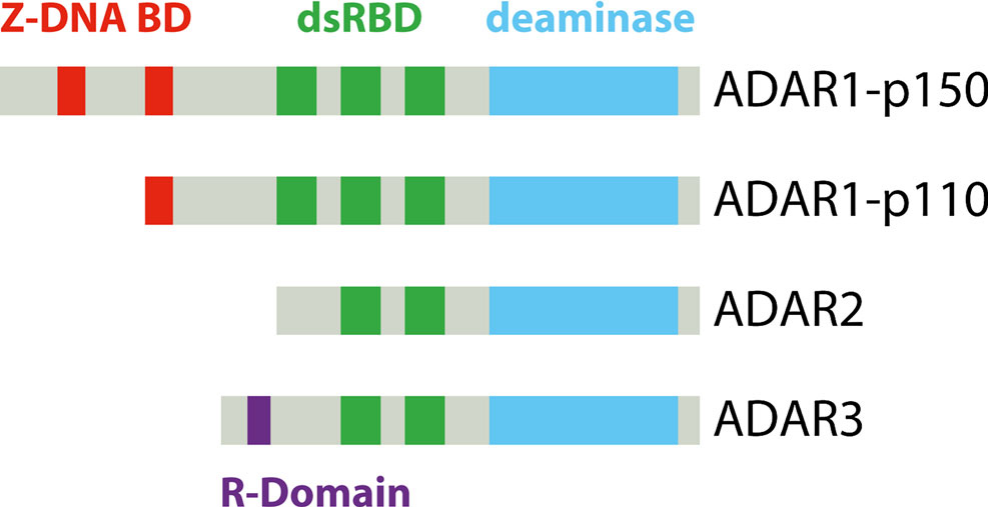
The ADAR protein family. Four different ADAR proteins have been identified in mammals. Here, the domain organization is shown. All ADAR proteins contain a deaminase domain (*light blue*) at the C-terminal end and a variable number of double-stranded RNA binding domains (dsRBDs, *green*). Z-DNA binding domains (*red*) are specific for ADAR1 isoforms, whereas the single-stranded RNA-binding R-domain (*purple*) is unique for ADAR3

All ADAR family members are structurally similar (Fig. 2). They contain two (ADAR2) or three (ADAR1) double-stranded RNA binding domains (dsRBDs) in the N-terminal part and a deaminase domain at the C-terminus (Nishikura 2010). In addition, ADAR1-p150 harbors two Z-DNA binding domains at the N-terminal end, whereas ADAR1-p110 only harbors one Z-DNA domain (Athanasiadis et al. 2005; Schade et al. 1999). ADAR2 shows no N-terminal extension while ADAR3 contains an arginine-rich domain at the N-terminal end (Chen et al. 2000). It has been proposed that ADAR1 and ADAR2 act as homodimers but also heterodimer-formation has been observed (Chilibeck et al. 2006; Cho et al. 2003). For an in-depth review of the ADAR enzyme family, domain organization, and protein function, we would like to point the reader to the review by George et al. (2011).

## Site-specific versus promiscuous editing and conservation of editing

With about two million identified editing sites, it is a challenge to focus on sites that are exclusively relevant to a specific phenomenon. For instance, several studies have suggested that certain repetitive structures (Alu elements) act as binding platforms or baits for ADARs, increase the local concentration of the proteins, and thereby ultimately increase the editing frequency of sites in the vicinity of the repetitive element (Daniel et al. 2014; Daniel et al. 2012). Thus, in this case, editing within the repetitive sequence might only be a side effect of ADAR binding, but itself not be of physiological relevance. Therefore, it may be useful to classify A to I editing sites in order to focus on subsets of sites. Here, one can follow different criteria: For instance, a distinction between site-selective and promiscuously or hyperedited sites appears very useful (Wahlstedt and Ohman 2011). Site-selective sites often lie within coding regions whereas promiscuous sites are typically found in repeat-rich regions that primarily reside in noncoding parts of a given transcript. Site-selective events are typically located at highly conserved positions within transcripts and edited with higher frequencies. Moreover, it has been shown that at least the ADAR2 protein binds site-selective sites with higher affinity compared to promiscuously edited sites (Klaue et al. 2003). Promiscuously edited sites, in contrast, typically occur in clusters and frequently represent the length of a repetitive element that folds back on itself. In most cases, editing frequencies are lower at promiscuously edited sites versus site-selective sites. Nevertheless, promiscuous editing events might be still conserved, but conservation is typically much lower compared to site-selective sites (Pinto et al. 2014).

Obviously, conservation is another category to classify editing sites. Many editing sites cluster into species-specific repeat regions. For instance, the majority of human RNA-editing sites is located in Alu elements (Ramaswami et al. 2012). Therefore, mice do not share these editing sites with humans since Alu-elements are primate specific. Instead, the mouse genome contains other classes of repetitive elements like B1 and B2 SINEs (short interspersed nuclear elements), which are not found in humans. Consequently, most editing sites in mice cluster in B1 or B2 elements (Danecek et al. 2012). Only a small number of sites—mostly located in protein-coding regions—are conserved between mouse and human (Li et al. 2009). Pinto and colleagues have thoroughly addressed mammalian editing sites and defined a set of conserved sites throughout the mammalian clade (Pinto et al. 2014). They identify a set of 59 sites conserved between mice and humans. Moreover, almost all of these sites conserved between mouse and human are also edited in cattle and rat. Generally, conserved sites have higher editing levels, frequently locate to exonic sequences, and lie mostly within genes associated with the central nervous system. Interestingly, the authors also identify 17 sites that are highly conserved but locate to introns (Pinto et al. 2014). These might be interesting sites for further studies as an obvious reason for their conservation is lacking. Finally, most conserved sites exhibit similar editing levels in mouse and human, arguing for physiological importance of this tight regulation.

## A to I editing and its impact on coding targets

The work of several laboratories has allowed identification of a few hundred editing sites in protein-coding regions. However, as A to I editing has been first established for transcripts expressed in the central nervous system, we will first highlight recoding events in two well-studied, brain-specific targets: the *Gria2* (GluR-B) substrate coding for the glutamate receptor subunit B and the transcript coding for serotonin 2C receptor (HTR2C). In the *Gria2* substrate, A to I editing leads to recoding at two different sites that either affect desensitization kinetics of the receptor (R/G site) (Lomeli et al. 1994) or regulate permeability of the ion channel (Q/R site) (Hume et al. 1991; Verdoorn et al. 1991). Lack of editing at the Q/R site leads to epileptic seizures and death in mice (Higuchi et al. 2000) and has been associated with human diseases like amyotrophic lateral sclerosis (ALS) or malignant gliomas (Kawahara et al. 2004; Kwak and Kawahara 2005; Maas et al. 2001; Takuma et al. 1999). When a *Gria*2 pre-mRNA, constitutively edited at the Q/R site is expressed in ADAR2 null mice, lethality is rescued, suggesting that the *Gria2* Q/R site is the major substrate for ADAR2 (Higuchi et al. 2000).

*HTR2C* encodes the serotonin receptor. Here, editing takes place at five sites in exon 5, which yields up to 24 different protein isoforms and modulates protein interaction, desensitization, and trafficking of HTR2C isoforms (Burns et al. 1997; Marion et al. 2004). Interestingly, mice with altered editing of the serotonin 2C receptor mRNA exhibit characteristics of the Prader-Willi syndrome, suggesting that editing of the pre-mRNA is crucial (Morabito et al. 2010). Besides the *Gria2* and *HTR2C* genes, A to I editing events leading to amino acid exchanges have been characterized for a number of protein-coding genes: Editing of the *NEIL1* pre-mRNA, for instance, leads to an arginine to lysine exchange and modulates the lesion specificity of the NEIL1 DNA repair enzyme (Yeo et al. 2010). AZIN1 editing, on the other hand, has been linked to hepatocellular carcinoma (Chen et al. 2013). Editing of the voltage-gated potassium channel K_V_1.1 affects recovery from inactivation (Bhalla et al. 2004). For a more thorough review regarding protein-coding targets, we would like to redirect the reader to two review articles from our group: (Pullirsch and Jantsch 2010; Tariq and Jantsch 2012).

Clearly, A to I editing plays a crucial role in recoding brain-specific transcripts. This might potentially reflect the need for increased diversity of neuronal ion channels and receptors. However, comparison of editing levels in the *FlnA* transcript in different mouse tissues has shown very high editing levels in the stomach, lung, or large intestine. In contrast, editing levels in brain regions like cortex or cerebellum are only moderate (Stulic and Jantsch 2013). In accordance with previous data (Wahlstedt et al. 2009), the editing levels of FlnA were also found to increase during development and reach highest levels in adult tissues. Therefore, these data suggest that A to I editing in coding regions might not only affect brain-specific targets, but also have a previously unappreciated impact outside the nervous system.

When comparing the occurrence of A to I editing sites in invertebrates and vertebrates, a strong shift of editing sites from protein-coding regions to nonprotein-coding regions of the transcriptome becomes evident. St Laurent et al. identified several hundred conserved editing sites leading to amino acid exchanges suggesting a widespread role of editing in *Drosophila* (St Laurent et al. 2013). In comparison, the number of conserved nonsynonymous editing sites is strongly decreased in mammals where only about 50 conserved editing sites are known (Pinto et al. 2014). Moreover, a very recent deep-sequencing study revealed 57,108 recoding sites in the squid nervous system (Alon et al. 2015). This clearly demonstrates the importance of mRNA-recoding by A to I editing in *Drosophila* and squid, suggesting that nonsynonymous A to I editing may be more important for invertebrate species compared to mammals, where editing is more dominant in noncoding parts of the transcriptome (Peng et al. 2012; Ramaswami et al. 2012).

## A to I RNA-editing in Alu elements

**Fig. 3:**
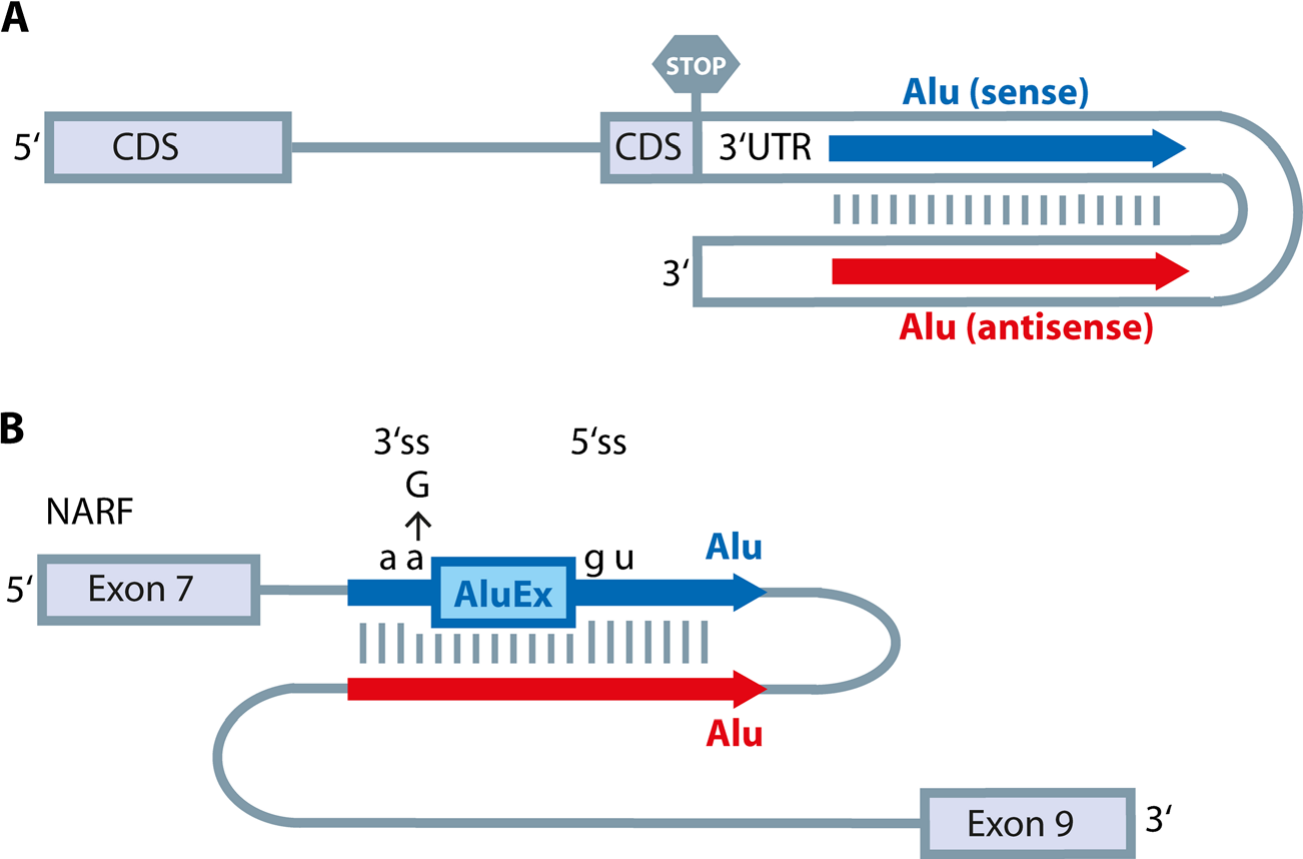
Alu elements and their role in A to I editing. **a** Alu elements frequently reside in noncoding regions of genes (e.g., 3′ UTRs). If two Alu elements (depicted in *blue and red*) are located in inverted orientation, they can form double-stranded structures and therefore be targeted by ADAR proteins. CDS = protein coding sequence. **b** Alu elements may also integrate into intronic regions. As shown for the NARF pre-mRNA, two Alus form double-stranded structures and therefore are edited. Editing leads to creation of an additional 3′ splice site (3′ss) and thereby an alternative exon (AluEx) is created using an already existing 5′ splice site (5′ss)

More than 90 % of editing in the human transcriptome occurs in Alu elements (Athanasiadis et al. 2004; Bazak et al. 2014a, b; Levanon et al. 2004). Alu elements are conserved, ~300 nucleotide long repeats that belong to the SINE family of retrotransposons found abundantly in primate genomes. Alu elements are not distributed randomly in the genome (Cordaux and Batzer 2009). They are enriched in gene-rich regions, where they are located within noncoding segments of transcripts, such as introns and untranslated regions (Versteeg et al. 2003). It was shown that editing is favored when two Alu elements in genes are located in inverted orientation and their distance is shorter than 2 kb (Fig. 3a). This observation suggests that these Alu elements can form double-stranded structures and therefore are a substrate for ADAR editing (Athanasiadis et al. 2004). In a recent study, Bazak and colleagues studied the features that contribute to the “editability” of Alu elements on a genome-wide scale (Bazak et al. 2014b). They confirm that the distance between two adjacent inverted Alu elements is the most important feature: short distances between two Alu elements increase the editability, and the distance alone accounts for about 30 % of the variability in Alu editing. Other factors such as the length of Alu elements, Alu subfamiliy, and sequence identity only add minor variability. Moreover, editability is higher if both Alu elements reside in the same exon or intron. However, all investigated factors only contribute to about 1/3 of editing variability. This indicates that other features specific to the context of individual Alus are important as well.

The role of widespread Alu RNA-editing is not well understood. However, a number of studies have been conducted to shed light on this aspect of A to I editing. It has been proposed that edited Alu elements can regulate mRNA expression. Several independent studies have shown that inverted Alu elements in the 3′UTRs of mRNAs strongly repress gene expression. However, the underlying molecular mechanisms seem not fully elucidated, and different pathways have been proposed to explain the reduction (Capshew et al. 2012; Chen et al. 2008). For instance, it was proposed that highly edited Alu elements bind to p54nrb, an RNA-binding protein showing high preference for inosine-containing RNAs. Binding of p54nrb, in turn, would prevent mRNA export to the cytoplasm (Chen and Carmichael 2008; Chen et al. 2008; Hu et al. 2015). Also, inverted Alu elements, highly edited in this case, may present a platform for the recruitment of RNA binding proteins. Therefore, inverted Alu elements in mRNAs - edited or nonedited - might serve as a platform for dsRNA binding proteins that modulate mRNA localization, translation, processing, or modification (Prasanth et al. 2005).

A major effect of Alu elements on the primate transcriptome is the introduction of new exons in existing mRNAs (Schmitz and Brosius 2011). Alu elements consist of two arms separated by poly(A) sequences. When they are inserted in the gene in antisense orientation, the poly(A) sequence is transcribed as a poly(U) tract that potentially acts as a polypyrimidine tract and might change splice patterns (Deininger 2011). Moreover, there are 9 potential 5′ splice sites and 14 potential 3′ splice sites located within the consensus sequence of Alu elements. A few mutations within the potential 3′ or 5′ splice sites are sufficient to create a new exon. Similarly, editing in inverted Alu sequences can promote the exonization of Alu elements in the transcriptome. Indeed, it has been shown that editing can create new splice sites (Sela et al. 2007; Sorek et al. 2002). For example, nuclear prelamin A recognition factor (NARF) has an Alu-exon which is regulated by editing (Lev-Maor et al. 2007). Here, RNA-editing can create a 3′ splice site (Fig. 3b) and also eliminates a premature stop codon within the Alu-exon. It has been suggested that 5 % of all alternatively spliced exons were originally derived from Alu elements (Sorek et al. 2002). Therefore, Alu elements and RNA-editing within them may increase transcriptome variation and accelerate primate evolution. However, in most cases, Alu exonization disrupts the transcript structure and affects protein function (Varon et al. 2003). Recently, a protection mechanism against Alu exonization and the production of aberrant mRNAs was proposed (Zarnack et al. 2013). It was shown that the RNA binding protein hnRNP C can bind cryptic splice sites in Alus and thereby prevent recognition of Alu elements by the splicing factor U2AF65.

In sum, editing in Alu elements clearly contributes to transcriptome diversity. Most importantly, Alu elements are primate specific, and many of the editing sites in Alu elements only occurred very recently in the evolution of the great ape lineage (Ramaswami et al. 2013). Thus, it is tempting to speculate that Alu elements themselves and editing in Alu elements are major driving forces for human evolution (Levanon and Eisenberg 2015).

## Two intrinsically coupled RNA processing events: pre-mRNA splicing and A to I editing

Both adenosine to inosine editing and alternative splicing contribute to diversification of mammalian transcriptomes and dramatically increase the number of transcript isoforms that can be generated from a given gene. Interestingly, ADAR1 and ADAR2 have been found associated with spliceosomal proteins (Raitskin et al. 2001). Similar to other posttranscriptional processes, mRNA-splicing is coordinated with transcription via the C-terminal domain of RNA-polymerase II (pol-II CTD) (Maniatis and Reed 2002). Therefore, it seems likely that A to I editing—per definition a nuclear, posttranscriptional mRNA processing step—might be integrated with other processing steps following transcription in a similar way. Indeed, it has been shown that the CTD is essential for efficient auto-editing of the ADAR2 pre-mRNA (Laurencikiene et al. 2006). The observation that both exonic editing sites in the *Gria2* transcript lie close to 5′ splice sites has risen the notion that editing at these sites might be linked to splicing. The so-called R/G editing site in *Gria2* is located at position −2 (relative to the next downstream exon-intron boundary). Editing is directed to this site by base-pairing of the region surrounding the editing site with a base-complementary region, located in the next downstream intron, called the editing complementary site (ECS). Binding of ADARs but also the base-pairing of the ECS with the site surrounding the edited adenosine might therefore interfere with base-pairing of the spliceosomal U1 snRNA which needs to access the 5′ splice site. Moreover, once edited, the inosine located at position −2 may also interfere with the base-pairing of U1 snRNA (Schoft et al. 2007). In all cases where the double-stranded structure required to define the editing site is generated by the basepairing of intronic and exonic sequences, editing must happen prior to splicing since the ECS required for ADAR targeting is located within the intron (Higuchi et al. 1993). Thus, removal of intronic ECSs by splicing will prevent editing and thus control the extent of editing. Consistently, an RNA-seq approach to determine editing levels in nascent RNA suggested that editing occurs cotranscriptionally before the bulk of introns has been removed (Rodriguez et al. 2012).

Initial evidence that editing can indeed influence splicing comes from two observations: In the ADAR2 knockout mouse, the ratio of the stubstrate *Gria2* pre-mRNA versus mature RNA is shifted. Levels of *Gria2* pre-mRNA increase in the knockout mouse whereas mRNA levels drop (Higuchi et al. 2000). In addition, it has been reported that aberrant editing in *Drosophila* leads to exon skipping in the *para* transcript (Reenan et al. 2000). Consequently, Bratt and Öhman determined how editing and splicing interact at the *Gria2* R/G site. They show clear interference of both processes. Apparently, the stem required for ADAR2 binding reduces splicing efficiency in vitro, but does not affect splicing in vivo (Bratt and Ohman 2003). More recent data even suggest that editing itself can reduce splicing of intron 13 in *Gria2* and thereby affect a downstream alternative splicing event (Penn and Greger 2009; Schoft et al. 2007). Here, it was proposed that the pol-II CTD enhances editing at the R/G site by inhibiting splicing of the adjacent intron in order to ensure that the editing competent stem formed between exon and intron is preserved (Ryman et al. 2007).

**Fig. 4:**
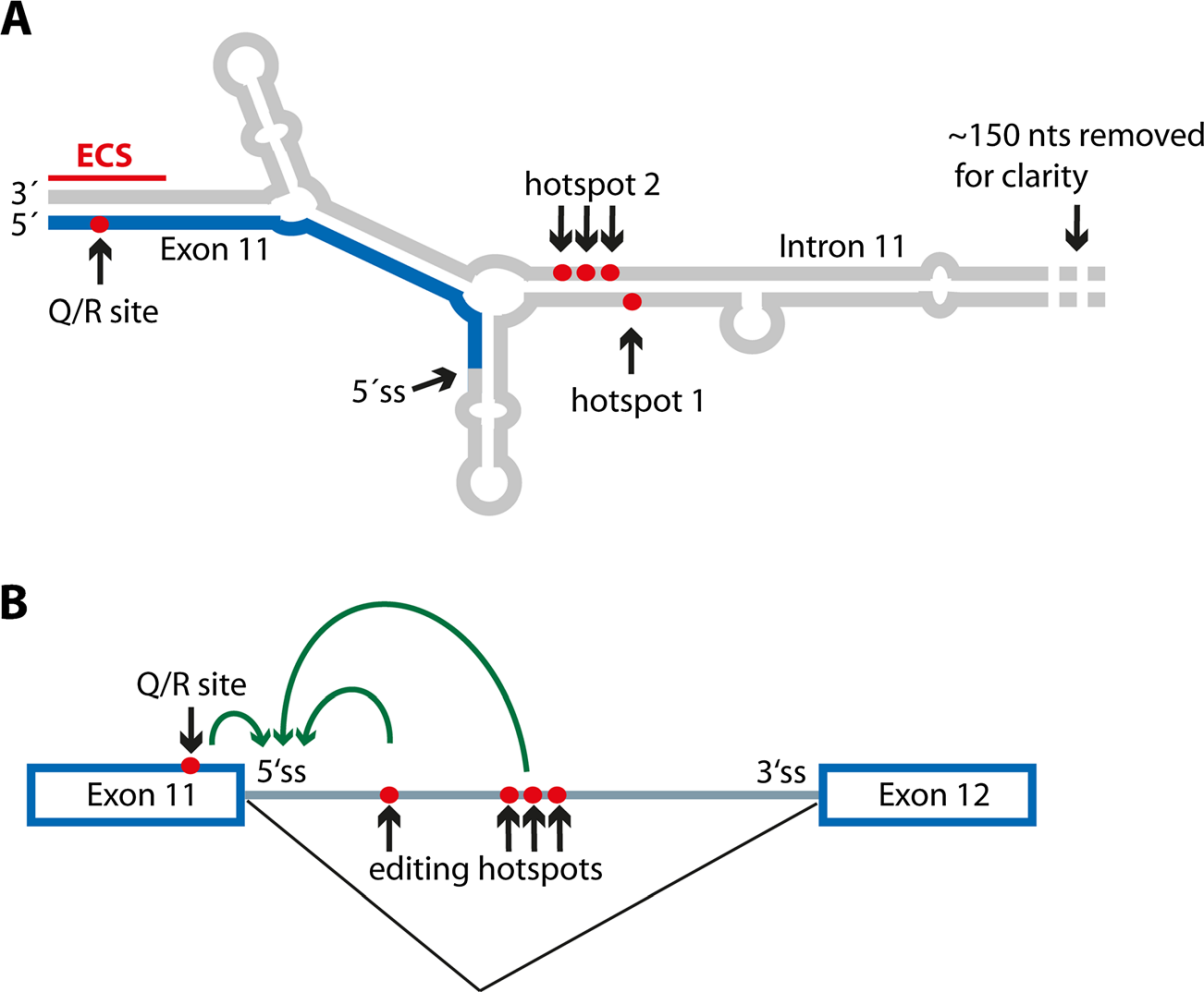
Tight regulation of A to I editing and mRNA splicing at the Gria2 Q/R site. **a** The Q/R editing site in exon 11 (*blue*) forms an editing competent stem with the downstream intron 11 (*gray*). Two additional editing hotspots are located in the intron. Editing sites are marked by *red dots*. ECS = editing complementary site. **b** Editing at hotspots 1 and 2 has to take place in order to allow efficient removal of intron 11 by splicing. Apparently, intronic editing acts as a safe-guard to ensure efficient editing at the Q/R site in exon 11. For details, please refer to the text. Editing sites are marked by *red dots. Green arrows* indicate that editing enhances splicing at the 5’ss

Still, events at the second editing site in the *Gria2* transcript are most interesting. As mentioned, Q/R site editing in *Gria2* is essential for viability (Higuchi et al. 2000). In adult mice, editing at this site reaches almost 100 % in the mature mRNA. Editing at the Q/R site in exon 11 is also accompanied by editing events in the adjacent intron 11, clustering at positions +60 and +262-264 relative to the Q/R site, called hotspot 1 and hotspot 2 (Fig. 4a) (Higuchi et al. 1993). Editing at these hotspots in intron 11 of the *Gria2* pre-mRNA is required for efficient intron removal and thus export of the mRNA to the cytoplasm (Fig. 4b) (Penn et al. 2013; Schoft et al. 2007). Editing at the intronic hotspots 1 and 2 might therefore be a control mechanism for efficient Q/R site editing in the mature mRNA. Intronic (and exonic) editing events ensure that only edited pre-mRNA is subjected to splicing, underlining the importance of this particular editing event (Penn et al. 2013; Schoft et al. 2007). Consistently, Penn and colleagues show that after knockdown of ADAR2 in cultured neurons, *Gria2* Q/R site editing remains unaffected, suggesting that the “safe-guard” mechanism can efficiently compensate for varying ADAR2 levels (Penn et al. 2013). In addition, the pol-II CTD apparently inhibits excision of intron 11 downstream of the Q/R site and thereby helps to ensure that editing precedes splicing (Ryman et al. 2007).

**Fig. 5:**
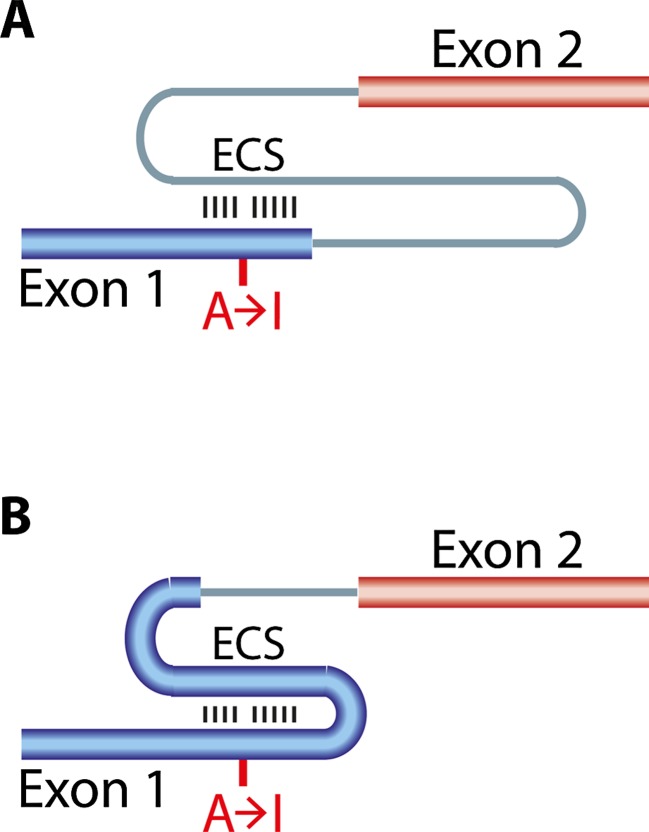
A double-stranded RNA structure is required for ADAR binding. **a** The double-stranded structure is frequently formed between exons (first exon depicted in *blue*, second in *red*) and introns, **b** but can also be formed within exons. ECS = editing complementary site

Base-pairing between exonic and intronic sequences also has been shown to regulate alternative splicing and editing of the HTR2C pre-mRNA (Flomen et al. 2004; Grohmann et al. 2010) (Fig. 5a). Moreover, in the ADAR2 pre-mRNA, an intronic AA dinucleotide can be subjected to editing and converted to A(I), and subsequently recognized by the splicing machinery as the terminal AG dinucleotide of a 3′ splice site (Rueter et al. 1999). Ultimately, this alternative splicing event results in the inclusion of another 47 nucleotides into the mRNA that leads to a frame shift causing premature termination of translation. This, in turn, autoregulates the levels of active ADAR2 in the cell (Rueter et al. 1999). Editing may not only influence splicing events in close proximity. Indeed, Agrawal and Stormo provide evidence that editing efficiency correlates with a distant downstream alternative splicing event in *Drosophila* (Agrawal and Stormo 2005).

Finally, a series of studies aimed at unravelling global interactions between editing and splicing. Solomon et al. observed changes in alternative splicing upon knockdown of ADAR1 in human cell lines (Solomon et al. 2013). However, many changes in splicing could not be linked to nearby editing events. Instead, it appeared that A to I editing was modulating *trans*-acting factors involved in the splicing process (Solomon et al. 2013). Similarly, a comprehensive analysis of A to I editing in *Drosophila* suggests that editing might promote alternative splicing by targeting transcripts that code for RNA binding proteins (St Laurent et al. 2013). Interestingly, Nova1—a brain-specific alternative splicing factor—can be edited, and evidence suggests that editing leads to increased protein half-life (Irimia et al. 2012). Additionally, Nova1 itself affects alternative splicing of several edited transcripts (Irimia et al. 2012) thus supporting the *Drosophila* data (St Laurent et al. 2013). Still, St Laurent et al. show that edited transcripts exhibit more complex alternative splicing patterns compared to transcripts that are not edited. However, when comparing wild-type and *Drosophila* ADAR null flies, this ratio did not change. Thus, the increase in editing in transcripts undergoing complex alternative splicing might rather be the consequence of alternative splicing and not the cause for alternative splicing. An earlier study supports this result and suggests that alternatively spliced exons are edited with higher frequency (Rodriguez et al. 2012). In sum, the global approaches that addressed the connection between editing and splicing support the general view that editing can lead to recoding in RNA binding proteins and thereby indirectly cause alternative splicing events. Vice versa, changes in (alternative) splicing might also lead to changes in editing frequencies.

## Regulation of A to I editing activity and factors that determine editing efficiency

**Fig. 6:**
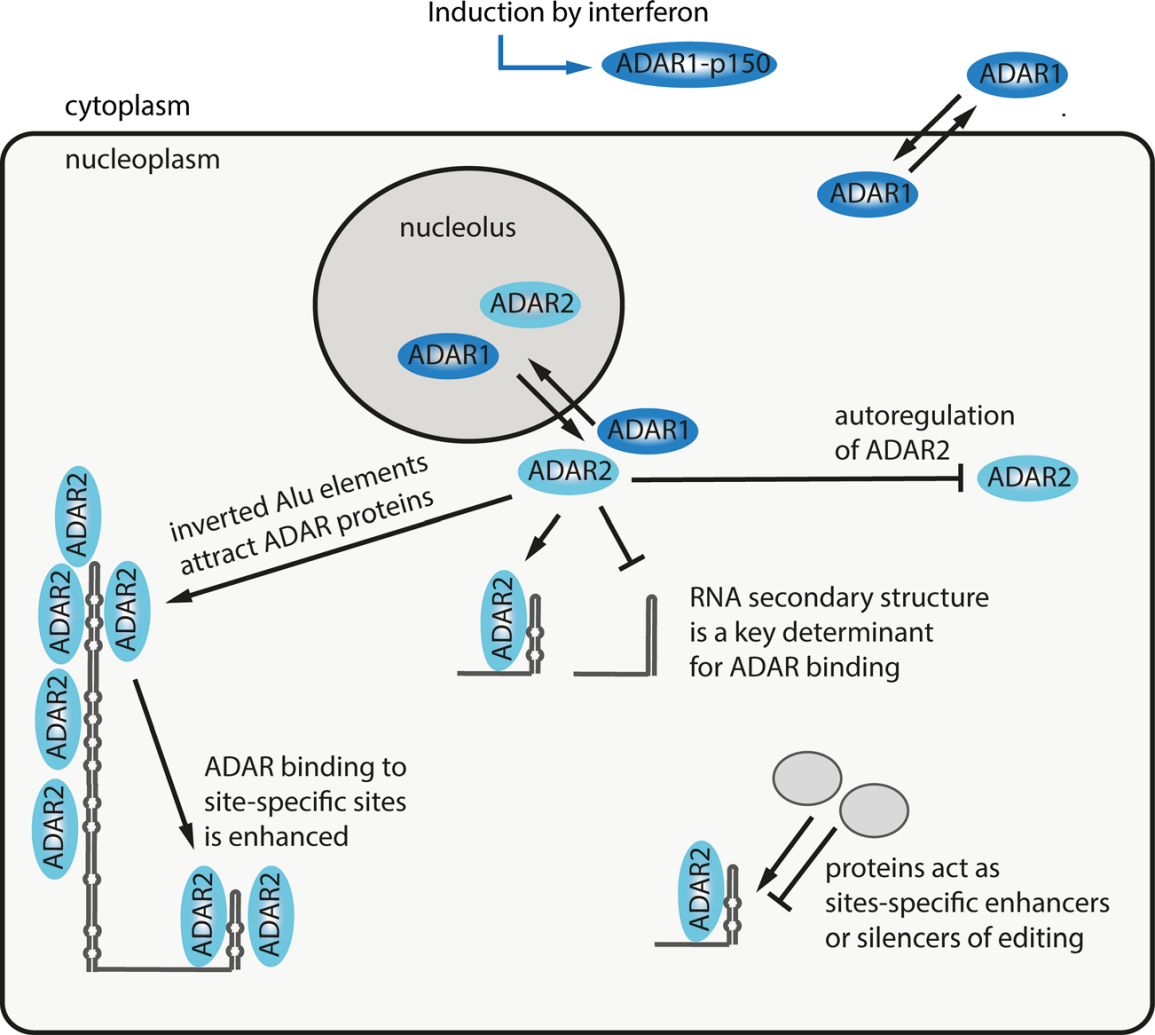
Factors that determine or regulate adenosine to inosine editing. The level of A to I editing is determined by a series of factors: The dominant factor is the RNA structure itself. Besides this, the subcellular distribution of ADAR proteins certainly contributes to the extent of editing. Moreover, proteins have been identified that regulate editing in a site-specific manner. Finally, induction of ADAR1-p150 by interferon most likely upregulates the extent of editing

Editing levels in coding and noncoding regions of mRNAs range from barely detectable to almost 100 % (Li et al. 2009). Moreover, editing frequencies substantially differ in various tissues and generally increase during development (Stulic and Jantsch 2013; Wahlstedt et al. 2009). Interestingly, ADAR protein levels are not increased accordingly and stay relatively constant during development as seen by immunoblotting (Wahlstedt et al. 2009). Thus, differences in expression of the deaminase cannot explain the increase of editing during development (Wahlstedt et al. 2009). Instead, these findings argue for a tight control of editing levels and suggest that other factors might regulate A to I editing (Fig. 6). Still, autoregulation of ADAR2 in mice, where editing of the ADAR2 pre-mRNA leads to a novel splice site that in turn generates a nonfunctional mRNA, represents a major mechanism to keep ADAR2 protein levels constant (Rueter et al. 1999). Loss of ADAR2 autoregulation leads to altered ADAR2 protein expression and significant changes in editing of several ADAR2 editing substrates (Feng et al. 2006). ADAR1 and ADAR2 enzymes are dynamically associated with the nucleolus (Desterro et al. 2003; Sansam et al. 2003). Both enzymes constantly shuttle between nucleolus and nucleoplasm. Upon expression of editing substrates, ADAR1 and ADAR2 delocalize from the nucleolus to the nucleoplasm (Desterro et al. 2003). This suggests that editing activity is regulated by shuttling of the proteins between nucleolus and nucleoplasm. Enhanced translocation of ADAR2 to the nucleoplasm results in increased editing of ADAR2 substrates (Sansam et al. 2003). The default localization to the nucleolus might also prevent ADAR enzymes from editing the “wrong” substrate. Interestingly, several lines of evidence suggest that small nucleolar RNAs (snoRNAs) play a role in regulating editing activity. The snoRNA MBII-52 matches a nucleotide tract in the HTR2C pre-mRNA and appears to specifically inhibit editing efficiency in the HTR2C transcript (Vitali et al. 2005). Moreover, in a mouse model that lacks expression of MBII-52, editing levels of the HTR2C transcript are significantly elevated, clearly demonstrating the contribution of MBII-52 to editing (Doe et al. 2009).

ADAR1 isoforms do not only shuttle between nucleolus and nucleoplasm but do also shuttle between nucleus and cytoplasm (Nie et al. 2004; Strehblow et al. 2002; Yang et al. 2003a, 2003b). Since ADAR enzymes primarily act on nuclear pre-mRNA, this phenomenon might also control nuclear editing activity. Moreover, a couple of protein factors have been implicated in regulating editing activity. The phosphorylation-dependent prolyl-isomerase Pin1 is a positive regulator of ADAR2 editing activity by enforcing its stabilization and localization to the nucleus (Marcucci et al. 2011). In Pin1-deficient mouse embryonic fibroblasts, ADAR2 mislocalizes to the cytoplasm resulting in underediting of *Gria2*. The E3 ubiquitin ligase WWP2 is a negative regulator of ADAR2 (Marcucci et al. 2011). It promotes ubiquitination and subsequent degradation of ADAR2. The proteins RPS14, SFRS9, and DDX15 act as site-specific repressors of editing (Tariq et al. 2013). For instance, RPS14 and SFRS9 negatively affect editing of the cyFIP2 transcript. Expression of RPS14, SFRS9, and DDX15 decreases during brain development. This observation might—at least in part—explain the increase of editing levels during development. DSS1/SHFM1 and hnRNP A2/B1 are additional regulators of editing (Garncarz et al. 2013). Moreover, at the transcriptional level, CREB1 enhances ADAR2 expression (Peng et al. 2006). Protein factors can also act indirectly via adding protein modifications: ADAR1 is a target of SUMO-1 modification, which reduces the editing activity in vitro (Desterro et al. 2005). A particularly interesting modulator of editing activity might be ADAR3. ADAR3 is believed to be catalytically inactive, but contains an RNA binding domain as well as a deaminase domain. ADAR3 has been tested on various editing substrates, and deamination activity has not been found (Chen et al. 2000; Schneider et al. 2014). Since ADAR3 is highly expressed in the brain and strongly binds double-stranded as well as single-stranded RNA, it may compete with ADAR2 and ADAR1 for substrate binding (Chen et al. 2000).

Since binding of ADAR proteins is determined by the RNA structure, differences in secondary structures can be an important factor for editing efficiency (Enstero et al. 2009; Tian et al. 2011). In the mRNA encoding Gabra3, the editing site is located within a stem exclusively formed by exon 9 (Ohlson et al. 2007). Nevertheless, an adjacent intronic stem structure has been reported to increase editing efficiency at the exonic site (Daniel et al. 2012). The authors suggest that the intronic stem acts as bait for ADAR2 and thereby increases the local concentration of the editing enzyme. Thereby, the editing efficiency of the nearby exonic site is increased. Since many similar intronic stem structures close to coding editing sites exist throughout the transcriptome, the authors speculate that this might be a general mode of action. A follow-up study supports this assumption and shows that Alu elements upstream of the Neil1 editing site stimulate editing of the exonic site. Similarly, other site-selective editing events are significantly enriched in the vicinity of Alu elements. Taken together, these findings suggest that Alu elments are an important driver for site-selective editing in primates (Daniel et al. 2014).

## New roles for ADAR1: modulation of innate immunity and circular RNA biogenesis

During the last couple of years, a fascinating new role for ADAR1 became evident. It appears that ADAR1 protects double-stranded parts of the transcriptome from being recognized as foreign/viral double-stranded RNA. Apparently, the innate immune system can distinguish viral RNA from cellular RNA by sensing inosine residues.

As mentioned, ADAR1-p150 localizes to the cytoplasm, and its expression is induced by interferon alpha and gamma linking this enzyme to inflammation (Patterson et al. 1995; Rabinovici et al. 2001). Consistently, the ratio of inosine containing mRNAs markedly increases upon systemic inflammation (Yang et al. 2003a, b). Using an induced deletion of ADAR1, it was shown that the enzyme is required in embryonic and adult hematopoietic stem cells (Hartner et al. 2009). Loss of ADAR1 leads to upregulation of type I and type II interferon-inducible transcripts. Thus, ADAR1 acts as a suppressor of interferon signaling and potentially protects the organism from interferon-induced damage. Interestingly, specific deletion of only the ADAR1-p150 isoform is sufficient to cause embryonic lethality and increased interferon signaling (Ward et al. 2011).

**Fig. 7:**
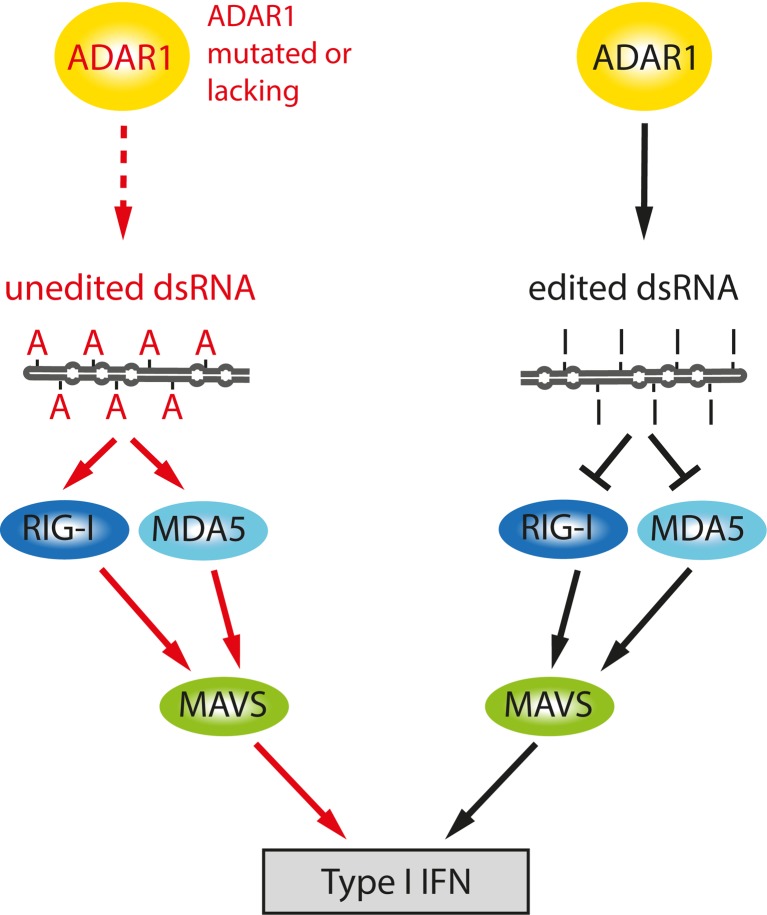
A to I editing and the innate immune response. The role of ADAR1 during the innate immune response is shown as proposed by Mannion and colleagues (Mannion et al. 2014). Loss of editing (for instance by mutations in ADAR1) leads to increased levels of unedited double-stranded RNA. The unedited RNA enhances the inflammatory response and acts via RIG-I or MDA5 and MAVS. Adapted from Mannion et al. (2014)

Vitali and Scadden could show that double-stranded RNA containing I-U base pairs suppresses the induction of interferon-stimulated genes (Vitali and Scadden 2010). In addition, I-U containing dsRNAs suppress the induction of IRF3, which is essential for the activation of interferon-stimulated genes and apoptosis. Finally, Mannion and colleagues could rescue the embryonic lethality of ADAR1 by a homozygous deletion of MAVS (Mannion et al. 2014). MAVS is an essential player in a cellular pathway that senses viral RNA and stimulates interferon signaling. This suggests that in the absence of A to I editing, endogenous substrates may stimulate the antiviral sensing machinery (Fig. 7). Consistently, transfection of inosine-containing dsRNA oligonucleotides into mouse embryonic fibroblasts derived from ADAR1 knockout mice reduces the interferon response (Mannion et al. 2014). Thus, ADAR1 is an essential player in the innate immune system that helps to discriminate cellular from viral dsRNA. Consistently, ADAR1 has been shown to act as both an antiviral as well as proviral factor. Seemingly, editing of viral RNAs may also mask them from the immune system (Samuel 2011).

An emerging role for ADAR1 is its antagonistic effect on the biogenesis of circular RNAs. Circular RNAs can be produced by “backsplicing,” ligation of 5′ and 3′ ends, or as intermediates of RNA processing reactions (Lasda and Parker 2014). Recently, the number of identified circular RNAs increased to a couple of thousands due to the combined efforts of several groups using tailored RNA-seq methods and bioinformatics (Ivanov et al. 2015; Jeck et al. 2013; Memczak et al. 2013; Rybak-Wolf et al. 2015; You et al. 2015). Circular RNAs have been found associated with intronic Alu elements, which can promote the circularization process when flanking circular RNA precursors in an inverted orientation (Jeck et al. 2013; Liang and Wilusz 2014; Zhang et al. 2014). Interestingly, a knockdown of ADAR1 specifically increases expression of circular RNAs, suggesting that ADAR1 antagonizes the process of circular RNA formation potentially by editing and destabilizing the dsRNA structures required for Alu-mediated circular RNA generation (Ivanov et al. 2015; Rybak-Wolf et al. 2015). Since A to I editing is particularly prominent in the brain and circular RNA expression is elevated in neuronal tissues as well, it is tempting to speculate that both processes regulate neuronal gene expression in a competitive manner (Rybak-Wolf et al. 2015; You et al. 2015). Clearly, the antagonistic effect of ADAR1 in circular RNA biogenesis but also the biology of circular RNAs itself needs to be explored more intensively and thus opens up interesting avenues for further research.

## Final remarks

A to I RNA-editing has been originally identified as a nonspecific unwinding activity of double-stranded RNAs (Bass and Weintraub 1987). Later, the protein was shown to target mRNAs encoding several brain-specific receptor proteins. About 25 years after these findings, the transcriptome-wide impact of A to I editing on transcriptome diversification and functional adaptation are firmly established with millions of identified editing sites. The consequences of A to I editing are diverse: They include recoding events, effects on splicing, and roles in the innate immune response. However, the functional consequences and the regulation of most of these sites remain dark matter. Therefore, genome-wide screens for function and regulation are necessary to elucidate the biological implication of the bulk of A to I editing.

## References

[CR1] Agrawal R, Stormo GD (2005). Editing efficiency of a Drosophila gene correlates with a distant splice site selection. RNA.

[CR2] Alon S, Garrett SC, Levanon EY, Olson S, Graveley BR, Rosenthal JJ, Eisenberg E (2015) The majority of transcripts in the squid nervous system are extensively recoded by A-to-I RNA editing. eLife 4 10.7554/eLife.05198 PMC4384741 25569156

[CR3] Athanasiadis A, Rich A, Maas S (2004). Widespread a-to-I RNA editing of Alu-containing mRNAs in the human transcriptome. PLoS Biol.

[CR4] Athanasiadis A, Placido D, Maas S, Brown BA, Lowenhaupt K, Rich A (2005). The crystal structure of the Zbeta domain of the RNA-editing enzyme ADAR1 reveals distinct conserved surfaces among Z-domains. J Mol Biol.

[CR5] Barbosa-Morais NL, Irimia M, Pan Q, Xiong HY, Gueroussov S, Lee LJ, Slobodeniuc V, Kutter C, Watt S, Colak R, Kim T, Misquitta-Ali CM, Wilson MD, Kim PM, Odom DT, Frey BJ, Blencowe BJ (2012). The evolutionary landscape of alternative splicing in vertebrate species. Science.

[CR6] Bass BL, Weintraub H (1987). A developmentally regulated activity that unwinds RNA duplexes. Cell.

[CR7] Bazak L, Haviv A, Barak M, Jacob-Hirsch J, Deng P, Zhang R, Isaacs FJ, Rechavi G, Li JB, Eisenberg E, Levanon EY (2014). A-to-I RNA editing occurs at over a hundred million genomic sites, located in a majority of human genes. Genome Res.

[CR8] Bazak L, Levanon EY, Eisenberg E (2014). Genome-wide analysis of Alu editability. Nucleic Acids Res.

[CR9] Bhalla T, Rosenthal JJ, Holmgren M, Reenan R (2004). Control of human potassium channel inactivation by editing of a small mRNA hairpin. Nat Struct Mol Biol.

[CR10] Blanc V, Davidson NO (2003). C-to-U RNA editing: mechanisms leading to genetic diversity. J Biol Chem.

[CR11] Blanc V, Davidson NO (2010). APOBEC-1-mediated RNA editing. Wires Syst Biol Med.

[CR12] Blow M, Futreal PA, Wooster R, Stratton MR (2004). A survey of RNA editing in human brain. Genome Res.

[CR13] Bratt E, Ohman M (2003). Coordination of editing and splicing of glutamate receptor pre-mRNA. RNA.

[CR14] Burns CM, Chu H, Rueter SM, Hutchinson LK, Canton H, SandersBush E, Emeson RB (1997). Regulation of serotonin-2C receptor G-protein coupling by RNA editing. Nature.

[CR15] Capshew CR, Dusenbury KL, Hundley HA (2012). Inverted Alu dsRNA structures do not affect localization but can alter translation efficiency of human mRNAs independent of RNA editing. Nucleic Acids Res.

[CR16] Chen LL, Carmichael GG (2008). Gene regulation by SINES and inosines: biological consequences of A-to-I editing of Alu element inverted repeats. Cell Cycle.

[CR17] Chen CX, Cho DS, Wang Q, Lai F, Carter KC, Nishikura K (2000). A third member of the RNA-specific adenosine deaminase gene family, ADAR3, contains both single- and double-stranded RNA binding domains. RNA.

[CR18] Chen LL, DeCerbo JN, Carmichael GG (2008). Alu element-mediated gene silencing. EMBO J.

[CR19] Chen L, Li Y, Lin CH, Chan TH, Chow RK, Song Y, Liu M, Yuan YF, Fu L, Kong KL, Qi L, Li Y, Zhang N, Tong AH, Kwong DL, Man K, Lo CM, Lok S, Tenen DG, Guan XY (2013). Recoding RNA editing of AZIN1 predisposes to hepatocellular carcinoma. Nat Med.

[CR20] Chilibeck KA, Wu T, Liang C, Schellenberg MJ, Gesner EM, Lynch JM, MacMillan AM (2006). FRET analysis of in vivo dimerization by RNA-editing enzymes. J Biol Chem.

[CR21] Cho DSC, Yang WD, Lee JT, Shiekhattar R, Murray JM, Nishikura K (2003). Requirement of dimerization for RNA editing activity of adenosine deaminases acting on RNA. J Biol Chem.

[CR22] Cordaux R, Batzer MA (2009). The impact of retrotransposons on human genome evolution. Nat Rev Genet.

[CR23] Danecek P, Nellaker C, McIntyre RE, Buendia-Buendia JE, Bumpstead S, Ponting CP, Flint J, Durbin R, Keane TM, Adams DJ (2012). High levels of RNA-editing site conservation amongst 15 laboratory mouse strains. Genome Biol.

[CR24] Daniel C, Veno MT, Ekdahl Y, Kjems J, Ohman M (2012). A distant cis acting intronic element induces site-selective RNA editing. Nucleic Acids Res.

[CR25] Daniel C, Silberberg G, Behm M, Ohman M (2014). Alu elements shape the primate transcriptome by cis-regulation of RNA editing. Genome Biol.

[CR26] Deininger P (2011). Alu elements: know the SINEs. Genome Biol.

[CR27] Desterro JM, Keegan LP, Lafarga M, Berciano MT, O'Connell M, Carmo-Fonseca M (2003). Dynamic association of RNA-editing enzymes with the nucleolus. J Cell Sci.

[CR28] Desterro JM, Keegan LP, Jaffray E, Hay RT, O'Connell MA, Carmo-Fonseca M (2005). SUMO-1 modification alters ADAR1 editing activity. Mol Biol Cell.

[CR29] Doe CM, Relkovic D, Garfield AS, Dalley JW, Theobald DE, Humby T, Wilkinson LS, Isles AR (2009). Loss of the imprinted snoRNA mbii-52 leads to increased 5htr2c pre-RNA editing and altered 5HT2CR-mediated behaviour. Hum Mol Genet.

[CR30] Enstero M, Daniel C, Wahlstedt H, Major F, Ohman M (2009). Recognition and coupling of A-to-I edited sites are determined by the tertiary structure of the RNA. Nucleic Acids Res.

[CR31] Feng Y, Sansam CL, Singh M, Emeson RB (2006). Altered RNA editing in mice lacking ADAR2 autoregulation. Mol Cell Biol.

[CR32] Flomen R, Knight J, Sham P, Kerwin R, Makoff A (2004). Evidence that RNA editing modulates splice site selection in the 5-HT2C receptor gene. Nucleic Acids Res.

[CR33] Garncarz W, Tariq A, Handl C, Pusch O, Jantsch MF (2013). A high-throughput screen to identify enhancers of ADAR-mediated RNA-editing. RNA Biol.

[CR34] George CX, Samuel CE (1999). Human RNA-specific adenosine deaminase ADAR1 transcripts possess alternative exon 1 structures that initiate from different promoters, one constitutively active and the other interferon inducible. Proc Natl Acad Sci U S A.

[CR35] George CX, Gan Z, Liu Y, Samuel CE (2011). Adenosine deaminases acting on RNA, RNA editing, and interferon action. J Interferon Cytokine Res.

[CR36] Grohmann M, Hammer P, Walther M, Paulmann N, Buttner A, Eisenmenger W, Baghai TC, Schule C, Rupprecht R, Bader M, Bondy B, Zill P, Priller J, Walther DJ (2010). Alternative splicing and extensive RNA editing of human TPH2 transcripts. PLoS One.

[CR37] Hartner JC, Schmittwolf C, Kispert A, Muller AM, Higuchi M, Seeburg PH (2004). Liver disintegration in the mouse embryo caused by deficiency in the RNA-editing enzyme ADAR1. J Biol Chem.

[CR38] Hartner JC, Walkley CR, Lu J, Orkin SH (2009). ADAR1 is essential for the maintenance of hematopoiesis and suppression of interferon signaling. Nat Immunol.

[CR39] Higuchi M, Single FN, Kohler M, Sommer B, Sprengel R, Seeburg PH (1993). Rna editing of ampa receptor subunit glur-B - a base-paired intron-exon structure determines position and efficiency. Cell.

[CR40] Higuchi M, Stefan M, Single FN, Hartner J, Rozov A, Burnashev N, Feldmeyer D, Sprengel R, Seeburg PH (2000). Point mutation in an AMPA receptor gene rescues lethality in mice deficient in the RNA-editing enzyme ADAR2. Nature.

[CR41] Hu SB, Xiang JF, Li X, Xu Y, Xue W, Huang M, Wong CC, Sagum CA, Bedford MT, Yang L, Cheng D, Chen LL (2015). Protein arginine methyltransferase CARM1 attenuates the paraspeckle-mediated nuclear retention of mRNAs containing IRAlus. Genes Dev.

[CR42] Hume RI, Dingledine R, Heinemann SF (1991). Identification of a site in glutamate receptor subunits that controls calcium permeability. Science.

[CR43] Hundley HA, Bass BL (2010). ADAR editing in double-stranded UTRs and other noncoding RNA sequences. Trends Biochem Sci.

[CR44] Irimia M, Denuc A, Ferran JL, Pernaute B, Puelles L, Roy SW, Garcia-Fernandez J, Marfany G (2012). Evolutionarily conserved A-to-I editing increases protein stability of the alternative splicing factor Nova1. RNA Biol.

[CR45] Ivanov A, Memczak S, Wyler E, Torti F, Porath HT, Orejuela MR, Piechotta M, Levanon EY, Landthaler M, Dieterich C, Rajewsky N (2015). Analysis of intron sequences reveals hallmarks of circular RNA biogenesis in animals. Cell Rep.

[CR46] Jeck WR, Sorrentino JA, Wang K, Slevin MK, Burd CE, Liu J, Marzluff WF, Sharpless NE (2013). Circular RNAs are abundant, conserved, and associated with ALU repeats. RNA.

[CR47] Kawahara Y, Ito K, Sun H, Aizawa H, Kanazawa I, Kwak S (2004). Glutamate receptors: RNA editing and death of motor neurons. Nature.

[CR48] Kim U, Wang Y, Sanford T, Zeng Y, Nishikura K (1994). Molecular-cloning of cdna for double-stranded-Rna adenosine-deaminase, a candidate enzyme for nuclear-Rna editing. Proc Natl Acad Sci U S A.

[CR49] Kim DD, Kim TT, Walsh T, Kobayashi Y, Matise TC, Buyske S, Gabriel A (2004). Widespread RNA editing of embedded alu elements in the human transcriptome. Genome Res.

[CR50] Kiran AM, O'Mahony JJ, Sanjeev K, Baranov PV (2013). Darned in 2013: inclusion of model organisms and linking with Wikipedia. Nucleic Acids Res.

[CR51] Klaue Y, Kallman AM, Bonin M, Nellen W, Ohman M (2003). Biochemical analysis and scanning force microscopy reveal productive and nonproductive ADAR2 binding to RNA substrates. RNA.

[CR52] Kwak S, Kawahara Y (2005). Deficient RNA editing of GluR2 and neuronal death in amyotropic lateral sclerosis. J Mol Med.

[CR53] Lasda E, Parker R (2014). Circular RNAs: diversity of form and function. RNA.

[CR54] Laurencikiene J, Kallman AM, Fong N, Bentley DL, Ohman M (2006). RNA editing and alternative splicing: the importance of co-transcriptional coordination. EMBO Rep.

[CR55] Levanon EY, Eisenberg E (2015). Does RNA editing compensate for Alu invasion of the primate genome?. BioEssays : News Rev Mol Cell Dev Biol.

[CR56] Levanon EY, Eisenberg E, Yelin R, Nemzer S, Hallegger M, Shemesh R, Fligelman ZY, Shoshan A, Pollock SR, Sztybel D, Olshansky M, Rechavi G, Jantsch MF (2004). Systematic identification of abundant A-to-I editing sites in the human transcriptome. Nat Biotechnol.

[CR57] Levanon EY, Hallegger M, Kinar Y, Shemesh R, Djinovic-Carugo K, Rechavi G, Jantsch MF, Eisenberg E (2005). Evolutionarily conserved human targets of adenosine to inosine RNA editing. Nucleic Acids Res.

[CR58] Lev-Maor G, Sorek R, Levanon EY, Paz N, Eisenberg E, Ast G (2007) RNA-editing-mediated exon evolution. Genome Biol 8 10.1186/gb-2007-8-2-r29 PMC1852406 17326827

[CR59] Li JB, Levanon EY, Yoon JK, Aach J, Xie B, Leproust E, Zhang K, Gao Y, Church GM (2009). Genome-wide identification of human RNA editing sites by parallel DNA capturing and sequencing. Science.

[CR60] Liang D, Wilusz JE (2014). Short intronic repeat sequences facilitate circular RNA production. Genes Dev.

[CR61] Licatalosi DD, Darnell RB (2010). RNA processing and its regulation: global insights into biological networks. Nat Rev Genet.

[CR62] Lomeli H, Mosbacher J, Melcher T, Hoger T, Geiger JR, Kuner T, Monyer H, Higuchi M, Bach A, Seeburg PH (1994). Control of kinetic properties of AMPA receptor channels by nuclear RNA editing. Science.

[CR63] Maas S, Melcher T, Herb A, Seeburg PH, Keller W, Krause S, Higuchi M, O'Connell MA (1996). Structural requirements for RNA editing in glutamate receptor pre-mRNAs by recombinant double-stranded RNA adenosine deaminase. J Biol Chem.

[CR64] Maas S, Patt S, Schrey M, Rich A (2001). Underediting of glutamate receptor GluR-B mRNA in malignant gliomas. Proc Natl Acad Sci U S A.

[CR65] Maniatis T, Reed R (2002). An extensive network of coupling among gene expression machines. Nature.

[CR66] Mannion NM, Greenwood SM, Young R, Cox S, Brindle J, Read D, Nellaker C, Vesely C, Ponting CP, McLaughlin PJ, Jantsch MF, Dorin J, Adams IR, Scadden AD, Ohman M, Keegan LP, O'Connell MA (2014). The RNA-editing enzyme ADAR1 controls innate immune responses to RNA. Cell Rep.

[CR67] Marcucci R, Brindle J, Paro S, Casadio A, Hempel S, Morrice N, Bisso A, Keegan LP, Del Sal G, O'Connell MA (2011). Pin1 and WWP2 regulate GluR2 Q/R site RNA editing by ADAR2 with opposing effects. EMBO J.

[CR68] Marion S, Weiner DM, Caron MG (2004). RNA editing induces variation in desensitization and trafficking of 5-hydroxytryptamine 2c receptor isoforms. J Biol Chem.

[CR69] Melcher T, Maas S, Herb A, Sprengel R, Seeburg PH, Higuchi M (1996). A mammalian RNA editing enzyme. Nature.

[CR70] Memczak S, Jens M, Elefsinioti A, Torti F, Krueger J, Rybak A, Maier L, Mackowiak SD, Gregersen LH, Munschauer M, Loewer A, Ziebold U, Landthaler M, Kocks C, le Noble F, Rajewsky N (2013). Circular RNAs are a large class of animal RNAs with regulatory potency. Nature.

[CR71] Morabito MV, Abbas AI, Hood JL, Kesterson RA, Jacobs MM, Kump DS, Hachey DL, Roth BL, Emeson RB (2010). Mice with altered serotonin 2C receptor RNA editing display characteristics of Prader-Willi syndrome. Neurobiol Dis.

[CR72] Morse DP, Aruscavage PJ, Bass BL (2002). RNA hairpins in noncoding regions of human brain and Caenorhabditis elegans mRNA are edited by adenosine deaminases that act on RNA. Proc Natl Acad Sci U S A.

[CR73] Nie Y, Zhao Q, Su Y, Yang JH (2004). Subcellular distribution of ADAR1 isoforms is synergistically determined by three nuclear discrimination signals and a regulatory motif. J Biol Chem.

[CR74] Nigita G, Veneziano D, Ferro A (2015). A-to-I RNA editing: current knowledge sources and computational approaches with special emphasis on on-coding RNA molecules. Front Bioeng Biotechnol.

[CR75] Nishikura K (2010). Functions and regulation of RNA editing by ADAR deaminases. Annu Rev Biochem.

[CR76] Ohlson J, Pedersen JS, Haussler D, Ohman M (2007). Editing modifies the GABA(A) receptor subunit alpha 3. RNA.

[CR77] Patterson JB, Samuel CE (1995). Expression and regulation by interferon of a double-stranded-RNA-specific adenosine deaminase from human cells: evidence for two forms of the deaminase. Mol Cell Biol.

[CR78] Patterson JB, Thomis DC, Hans SL, Samuel CE (1995). Mechanism of interferon action: double-stranded RNA-specific adenosine deaminase from human cells is inducible by alpha and gamma interferons. Virology.

[CR79] Peng PL, Zhong X, Tu W, Soundarapandian MM, Molner P, Zhu D, Lau L, Liu S, Liu F, Lu Y (2006). ADAR2-dependent RNA editing of AMPA receptor subunit GluR2 determines vulnerability of neurons in forebrain ischemia. Neuron.

[CR80] Peng Z, Cheng Y, Tan BC, Kang L, Tian Z, Zhu Y, Zhang W, Liang Y, Hu X, Tan X, Guo J, Dong Z, Bao L, Wang J (2012). Comprehensive analysis of RNA-Seq data reveals extensive RNA editing in a human transcriptome. Nat Biotechnol.

[CR81] Penn AC, Greger IH (2009). Sculpting AMPA receptor formation and function by alternative RNA processing. RNA Biol.

[CR82] Penn AC, Balik A, Greger IH (2013). Steric antisense inhibition of AMPA receptor Q/R editing reveals tight coupling to intronic editing sites and splicing. Nucleic Acids Res.

[CR83] Pinto Y, Cohen HY, Levanon EY (2014). Mammalian conserved ADAR targets comprise only a small fragment of the human editosome. Genome Biol.

[CR84] Porath HT, Carmi S, Levanon EY (2014). A genome-wide map of hyper-edited RNA reveals numerous new sites. Nat Commun.

[CR85] Prasanth KV, Prasanth SG, Xuan Z, Hearn S, Freier SM, Bennett CF, Zhang MQ, Spector DL (2005). Regulating gene expression through RNA nuclear retention. Cell.

[CR86] Pullirsch D, Jantsch MF (2010). Proteome diversification by adenosine to inosine RNA editing. RNA Biol.

[CR87] Rabinovici R, Kabir K, Chen M, Su Y, Zhang D, Luo X, Yang JH (2001). ADAR1 is involved in the development of microvascular lung injury. Circ Res.

[CR88] Raitskin O, Cho DS, Sperling J, Nishikura K, Sperling R (2001). RNA editing activity is associated with splicing factors in lnRNP particles: the nuclear pre-mRNA processing machinery. Proc Natl Acad Sci U S A.

[CR89] Ramaswami G, Li JB (2014). RADAR: a rigorously annotated database of A-to-I RNA editing. Nucleic Acids Res.

[CR90] Ramaswami G, Lin W, Piskol R, Tan MH, Davis C, Li JB (2012). Accurate identification of human Alu and non-Alu RNA editing sites. Nat Methods.

[CR91] Ramaswami G, Zhang R, Piskol R, Keegan LP, Deng P, O'Connell MA, Li JB (2013). Identifying RNA editing sites using RNA sequencing data alone. Nat Methods.

[CR92] Reenan RA, Hanrahan CJ, Ganetzky B (2000). The mle(napts) RNA helicase mutation in drosophila results in a splicing catastrophe of the para Na + channel transcript in a region of RNA editing. Neuron.

[CR93] Rodriguez J, Menet JS, Rosbash M (2012). Nascent-seq indicates widespread cotranscriptional RNA editing in Drosophila. Mol Cell.

[CR94] Rosenberg BR, Hamilton CE, Mwangi MM, Dewell S, Papavasiliou FN (2011) Transcriptome-wide sequencing reveals numerous APOBEC1 mRNA-editing targets in transcript 3′ UTRs. Nat Struct Mol Biol 10.1038/nsmb.1975 PMC3075553 21258325

[CR95] Rueter SM, Dawson TR, Emeson RB (1999). Regulation of alternative splicing by RNA editing. Nature.

[CR96] Rybak-Wolf A, Stottmeister C, Glazar P, Jens M, Pino N, Giusti S, Hanan M, Behm M, Bartok O, Ashwal-Fluss R, Herzog M, Schreyer L, Papavasileiou P, Ivanov A, Ohman M, Refojo D, Kadener S, Rajewsky N (2015). Circular RNAs in the mammalian brain are highly abundant, conserved, and dynamically expressed. Mol Cell.

[CR97] Ryman K, Fong N, Bratt E, Bentley DL, Ohman M (2007). The C-terminal domain of RNA Pol II helps ensure that editing precedes splicing of the GluR-B transcript. RNA.

[CR98] Sabin Leah R, Delás MJ, Hannon Gregory J (2013). Dogma derailed: the many influences of RNA on the genome. Mol Cell.

[CR99] Samuel CE (2011). Adenosine deaminases acting on RNA (ADARs) are both antiviral and proviral. Virology.

[CR100] Sansam CL, Wells KS, Emeson RB (2003). Modulation of RNA editing by functional nucleolar sequestration of ADAR2. Proc Natl Acad Sci U S A.

[CR101] Schade M, Behlke J, Lowenhaupt K, Herbert A, Rich A, Oschkinat H (1999). A 6 bp Z-DNA hairpin binds two Z alpha domains from the human RNA editing enzyme ADAR1. FEBS Lett.

[CR102] Schmitz J, Brosius J (2011). Exonization of transposed elements: a challenge and opportunity for evolution. Biochimie.

[CR103] Schneider MF, Wettengel J, Hoffmann PC, Stafforst T (2014). Optimal guideRNAs for re-directing deaminase activity of hADAR1 and hADAR2 in trans. Nucleic Acids Res.

[CR104] Schoft VK, Schopoff S, Jantsch MF (2007). Regulation of glutamate receptor B pre-mRNA splicing by RNA editing. Nucleic Acids Res.

[CR105] Sela N, Mersch B, Gal-Mark N, Lev-Maor G, Hotz-Wagenblatt A, Ast G (2007) Comparative analysis of transposed element insertion within human and mouse genomes reveals Alu's unique role in shaping the human transcriptome. Genome Biol 8 10.1186/gb-2007-8-6-r127 PMC2394776 17594509

[CR106] Sie CP, Kuchka M (2011). RNA editing adds flavor to complexity. Biochem Biokhimiia.

[CR107] Solomon O, Oren S, Safran M, Deshet-Unger N, Akiva P, Jacob-Hirsch J, Cesarkas K, Kabesa R, Amariglio N, Unger R, Rechavi G, Eyal E (2013). Global regulation of alternative splicing by adenosine deaminase acting on RNA (ADAR). RNA.

[CR108] Sorek R, Ast G, Graur D (2002). Alu-containing exons are alternatively spliced. Genome Res.

[CR109] St Laurent G, Tackett MR, Nechkin S, Shtokalo D, Antonets D, Savva YA, Maloney R, Kapranov P, Lawrence CE, Reenan RA (2013) Genome-wide analysis of A-to-I RNA editing by single-molecule sequencing in Drosophila. Nature Structural & Molecular Biology 10.1038/nsmb.2675 24077224

[CR110] Strehblow A, Hallegger M, Jantsch MF (2002). Nucleocytoplasmic distribution of human RNA-editing enzyme ADAR1 is modulated by double-stranded RNA-binding domains, a leucine-rich export signal, and a putative dimerization domain. Mol Biol Cell.

[CR111] Stulic M, Jantsch MF (2013). Spatio-temporal profiling of Filamin A RNA-editing reveals ADAR preferences and high editing levels outside neuronal tissues. RNA Biol.

[CR112] Szathmáry E, Jordán F, Pál C (2001). Can genes explain biological complexity?. Science.

[CR113] Takuma H, Kwak S, Yoshizawa T, Kanazawa I (1999). Reduction of GluR2 RNA editing, a molecular change that increases calcium influx through AMPA receptors, selective in the spinal ventral gray of patients with amyotrophic lateral sclerosis. Ann Neurol.

[CR114] Tariq A, Jantsch MF (2012). Transcript diversification in the nervous system: a to I RNA editing in CNS function and disease development. Front Neurosci.

[CR115] Tariq A, Garncarz W, Handl C, Balik A, Pusch O, Jantsch MF (2013). RNA-interacting proteins act as site-specific repressors of ADAR2-mediated RNA editing and fluctuate upon neuronal stimulation. Nucleic Acids Res.

[CR116] Tian N, Yang Y, Sachsenmaier N, Muggenhumer D, Bi J, Waldsich C, Jantsch MF, Jin Y (2011) A structural determinant required for RNA editing. Nucleic Acids Res 10.1093/nar/gkr144 PMC3141254 21427087

[CR117] Varon R, Gooding R, Steglich C, Marns L, Tang H, Angelicheva D, Yong KK, Ambrugger P, Reinhold A, Morar B, Baas F, Kwa M, Tournev I, Guerguelcheva V, Kremensky I, Lochmuller H, Mullner-Eidenbock A, Merlini L, Neumann L, Burger J, Walter M, Swoboda K, Thomas PK, von Moers A, Risch N, Kalaydjieva L (2003). Partial deficiency of the C-terminal-domain phosphatase of RNA polymerase II is associated with congenital cataracts facial dysmorphism neuropathy syndrome. Nat Genet.

[CR118] Verdoorn TA, Burnashev N, Monyer H, Seeburg PH, Sakmann B (1991). Structural determinants of ion flow through recombinant glutamate receptor channels. Science.

[CR119] Versteeg R, van Schaik BDC, van Batenburg MF, Roos M, Monajemi R, Caron H, Bussemaker HJ, van Kampen AHC (2003). The human transcriptome map reveals extremes in gene density, intron length, GC content, and repeat pattern for domains of highly and weakly expressed genes. Genome Res.

[CR120] Vitali P, Scadden AD (2010). Double-stranded RNAs containing multiple IU pairs are sufficient to suppress interferon induction and apoptosis. Nat Struct Mol Biol.

[CR121] Vitali P, Basyuk E, Le Meur E, Bertrand E, Muscatelli F, Cavaille J, Huttenhofer A (2005). ADAR2-mediated editing of RNA substrates in the nucleolus is inhibited by C/D small nucleolar RNAs. J Cell Biol.

[CR122] Wahlstedt H, Ohman M (2011). Site-selective versus promiscuous A-to-I editing. Wiley Interdiscip Rev RNA.

[CR123] Wahlstedt H, Daniel C, Enstero M, Ohman M (2009). Large-scale mRNA sequencing determines global regulation of RNA editing during brain development. Genome Res.

[CR124] Wang Q, Miyakoda M, Yang W, Khillan J, Stachura DL, Weiss MJ, Nishikura K (2004). Stress-induced apoptosis associated with null mutation of ADAR1 RNA editing deaminase gene. J Biol Chem.

[CR125] Ward SV, George CX, Welch MJ, Liou LY, Hahm B, Lewicki H, de la Torre JC, Samuel CE, Oldstone MB (2011). RNA editing enzyme adenosine deaminase is a restriction factor for controlling measles virus replication that also is required for embryogenesis. Proc Natl Acad Sci U S A.

[CR126] Yang JH, Luo X, Nie Y, Su Y, Zhao Q, Kabir K, Zhang D, Rabinovici R (2003). Widespread inosine-containing mRNA in lymphocytes regulated by ADAR1 in response to inflammation. Immunology.

[CR127] Yang JH, Nie Y, Zhao Q, Su Y, Pypaert M, Su H, Rabinovici R (2003). Intracellular localization of differentially regulated RNA-specific adenosine deaminase isoforms in inflammation. J Biol Chem.

[CR128] Yeo J, Goodman RA, Schirle NT, David SS, Beal PA (2010). RNA editing changes the lesion specificity for the DNA repair enzyme NEIL1. Proc Natl Acad Sci U S A.

[CR129] You X, Vlatkovic I, Babic A, Will T, Epstein I, Tushev G, Akbalik G, Wang M, Glock C, Quedenau C, Wang X, Hou J, Liu H, Sun W, Sambandan S, Chen T, Schuman EM, Chen W (2015). Neural circular RNAs are derived from synaptic genes and regulated by development and plasticity. Nat Neurosci.

[CR130] Zarnack K, Konig J, Tajnik M, Martincorena I, Eustermann S, Stevant I, Reyes A, Anders S, Luscombe NM, Ule J (2013). Direct competition between hnRNP C and U2AF65 protects the transcriptome from the exonization of Alu elements. Cell.

[CR131] Zhang XO, Wang HB, Zhang Y, Lu X, Chen LL, Yang L (2014). Complementary sequence-mediated exon circularization. Cell.

